# Three-dimensional infrared scanning: an enhanced approach for spatial registration of probes for neuroimaging

**DOI:** 10.1117/1.NPh.11.2.024309

**Published:** 2024-05-26

**Authors:** András Bálint, Christian Rummel, Marco Caversaccio, Stefan Weder

**Affiliations:** a University of Bern, ARTORG Center for Biomedical Engineering Research, Hearing Research Laboratory, Bern, Switzerland; b Inselspital, Bern University Hospital, University of Bern, Department of ENT - Head and Neck Surgery, Bern, Switzerland; c Inselspital, Bern University Hospital, University of Bern, University Institute of Diagnostic and Interventional Neuroradiology, Support Center for Advanced Neuroimaging (SCAN), Bern, Switzerland

**Keywords:** functional near-infrared spectroscopy, diffuse optical tomography, high density, electroencephalography, spatial accuracy, spatial registration

## Abstract

**Significance:**

Accurate spatial registration of probes (e.g., optodes and electrodes) for measurement of brain activity is a crucial aspect in many neuroimaging modalities. It may increase measurement precision and enable the transition from channel-based calculations to volumetric representations.

**Aim:**

This technical note evaluates the efficacy of a commercially available infrared three-dimensional (3D) scanner under actual experimental (or clinical) conditions and provides guidelines for its use.

**Method:**

We registered probe positions using an infrared 3D scanner and validated them against magnetic resonance imaging (MRI) scans on five volunteer participants.

**Results:**

Our analysis showed that with standard cap fixation, the average Euclidean distance of probe position among subjects could reach up to 43 mm, with an average distance of 15.25 mm [standard deviation (SD) = 8.0]. By contrast, the average distance between the infrared 3D scanner and the MRI-acquired positions was 5.69 mm (SD = 1.73), while the average difference between consecutive infrared 3D scans was 3.43 mm (SD = 1.62). The inter-optode distance, which was fixed at 30 mm, was measured as 29.28 mm (SD = 1.12) on the MRI and 29.43 mm (SD = 1.96) on infrared 3D scans. Our results demonstrate the high accuracy and reproducibility of the proposed spatial registration method, making it suitable for both functional near-infrared spectroscopy and electroencephalogram studies.

**Conclusions:**

The 3D infrared scanning technique for spatial registration of probes provides economic efficiency, simplicity, practicality, repeatability, and high accuracy, with potential benefits for a range of neuroimaging applications. We provide practical guidance on anonymization, labeling, and post-processing of acquired scans.

## 1 Introduction

In neuroimaging modalities, precise spatial alignment of probes (e.g., optodes and electrodes) on the scalp is pivotal. It may enhance spatial resolution, improve source estimation, strengthen between-subject reliability, and thus deepen our understanding of cerebral function.^1^ Conventionally, in the absence of spatial registration, the probes are positioned on the cranial surface following the international 10-20, 10-10, or 10-5 electroencephalogram (EEG) systems.^2^ These systems use relative positions that are individually determined based on the head circumference (i.e., the total distance from the inion to the nasion). To facilitate the probe mounting process, manufacturers offer an assortment of caps designed to accommodate a range of head circumferences, with typical increments of 2 cm. However, this method does not guarantee consistent coverage across different brain areas across participants and is subject to expected deviations in relative positioning.^3^ Moreover, even minor displacements can markedly impact the accuracy of source estimation.^1^ The challenges intensify when considering optical neuroimaging techniques, such as functional near-infrared spectroscopy (fNIRS) and high-density diffuse optical tomography. In a conventional fNIRS configuration, optical sources and detectors, referred to as optodes, are usually affixed to the scalp at an optimal distance of 30 mm, determined by the absorption characteristics of near-infrared light. Yet, the fixed distance between optodes renders it unfeasible to align the caps according to EEG systems, resulting in reduced anatomical consistency across participants with varying head sizes.^4^ To address these challenges, brain imaging studies necessitate a more accurate spatial registration methodology that is both anatomically precise and practical in terms of time and implementation. Traditional electromagnetic three-dimensional (3D) digitizers have been considered the gold standard for highly accurate spatial registrations.^5^ With the device, the separate probes can be marked on the head using an electromagnetic tracking method. However, their drawbacks include high costs, time-consuming consecutive probe registration, interference with medical implants (e.g., cochlear implants), and impracticality in certain populations.^6^^,^^7^ To address these limitations, less cost-intensive photogrammetric approaches gained popularity, utilizing a single camera for video recording around the head, enabling parallel acquisition of multiple probes and faster data collection.^7^^,^^8^ Yet, the computationally expensive and time-consuming reconstruction of 3D shapes from two-dimensional video frames poses challenges, limiting immediate assessment after recording.^6^ Adding a depth-sensing instrument (e.g., an infrared camera) to the setup offers multiple advantages. It facilitates quick 3D reconstruction, allowing immediate evaluation of the 3D mesh and avoiding additional experimental time due to lengthy reconstruction processes. Furthermore, the infrared camera is less sensitive to background information, allowing more effective recording under a variety of conditions. These advantages collectively make it the preferred choice for user-friendliness.

Despite these advantages, the efficacy of the system had not been validated under actual experimental (or clinical) conditions, nor were there clear guidelines for its use. Our goal was to fill these gaps.

In this technical note, we aim to evaluate the efficacy of a commercially available infrared 3D scanner as a potential solution for accurately localizing probes on the scalp of human participants. For evaluation, we used magnetic resonance imaging (MRI) scans as in Refs. 9 and 10. We also provide guidelines for data anonymization to promote data sharing among researchers and to ensure compliance with privacy and ethical standards. Finally, we provide the complete source code for anatomical mapping and integration into the MNE-Python library, a widely used neuroimaging data processing toolbox.^11^^,^^12^

## 2 Materials and Methods

The study followed the Declaration of Helsinki guidelines and was approved by the KEK-Bern (study number 2020-02978), and all participants provided written informed consent.

**Fig. 1:**
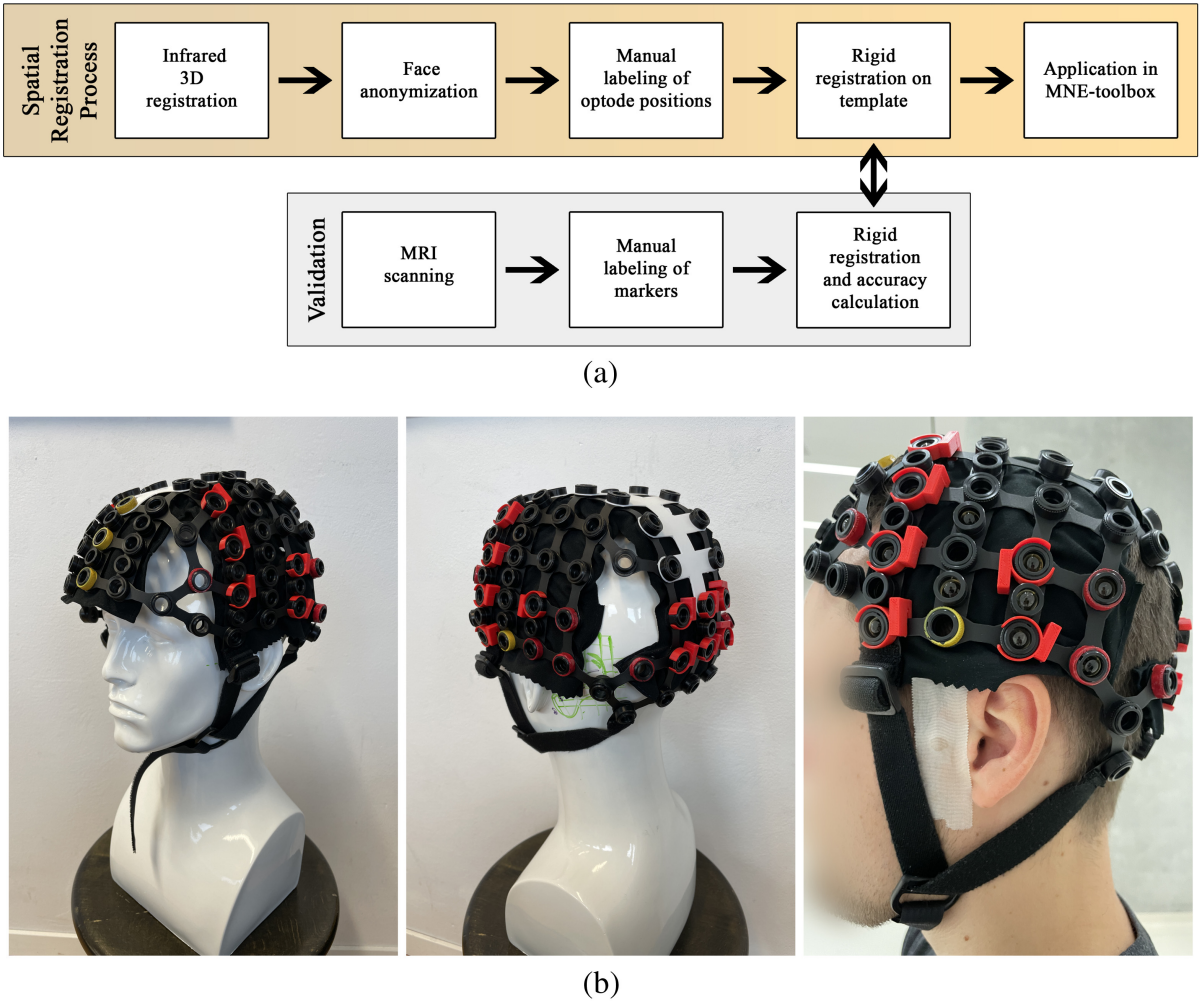
Study procedure. (a) Flowchart showing the different steps of our proposed 3D infrared scanning method (yellow box). We validated the method using MRI scanning (grey box). (b) We employed a custom-built fNIRS cap designed to evaluate accuracy across the temporal, visual, and prefrontal regions. To enhance detectability on infrared 3D scans, the holders were marked using colored 3D-printed markers or painted. Nitroglycerin markers were attached to the optode holders and anatomical landmarks, as depicted in the left image (captured after the MRI scan).

To estimate the spatial resolution, we registered probe positions using an infrared 3D registration method and performed MRI scans on five voluntary participants. We positioned an fNIRS optode holder cap on the participants’ heads and fixated nitroglycerin capsules inside the optode holders and on the nasion, left pre-auricular (LPA), and right pre-auricular (RPA) anatomical landmarks. The fNIRS cap used in this investigation is identical to that detailed in our published article.^13^ The cap is a custom assembly, constructed from the components provided in the manufacturer’s holder kit (Shimadzu, Kyoto, Japan). A total of 25 optodes were included in the study, spaced at an inter-optode distance of 30 mm. The configuration covers temporal, visual, and prefrontal brain regions. After mounting the cap, we performed an MRI scan on the participants. Subsequent to the MRI scan, the participants sat down on a chair, and we performed three successive infrared 3D scans under identical conditions [Fig. 1(a)].

### 2.1 fNIRS Cap Preparation and Infrared 3D Registration

For infrared 3D registration of the fNIRS cap, we suggest marking the optode holders with a distinct color to easily differentiate them on the acquired scans. In our case, we utilized red 3D-printed markers that could be fixated around the optode holders or painted the optode holders with nail polish [Fig. 1(b)].

For the spatial registration process, we utilized a depth-sensing infrared camera (Structure Sensor Pro, Occipital Inc., Boulder, Colorado, United States) attached to an iPad (iPad Pro 2020, Apple Inc., Los Altos, California, United States, iOS 14.3). To perform the registrations, we used the Scanner - Structure SDK (ver. 2.4.1) app. We configured the software settings suitable for our darkened room environment, with specifics outlined in Table 1. We moved the camera so that the participants’ heads were in the center of the screen (i.e., in the bounding box) and started the scan. We initiated the scanning from the participant’s front and slowly walked around them. When the software asked to stop to capture a key frame, we held still for a few seconds. Upon scan completion, we opted for the “Color View” and saved it locally in the default OBJ .zip format.

**Table 1:** Settings in the Scanner - Structure SDK app.

	Settings	Value
Streaming settings	Depth resolution	Full
	High-resolution color	On
	IR auto exposure	On
	IR analog gain	8×
SLAM, tracker, and mapper	Depth stream preset	Body
	SLAM option	Default
	Tracker type	Color + depth
	High-resolution mesh	On
	Improved mapper	On

### 2.2 Face Anonymization

**Fig. 2:**
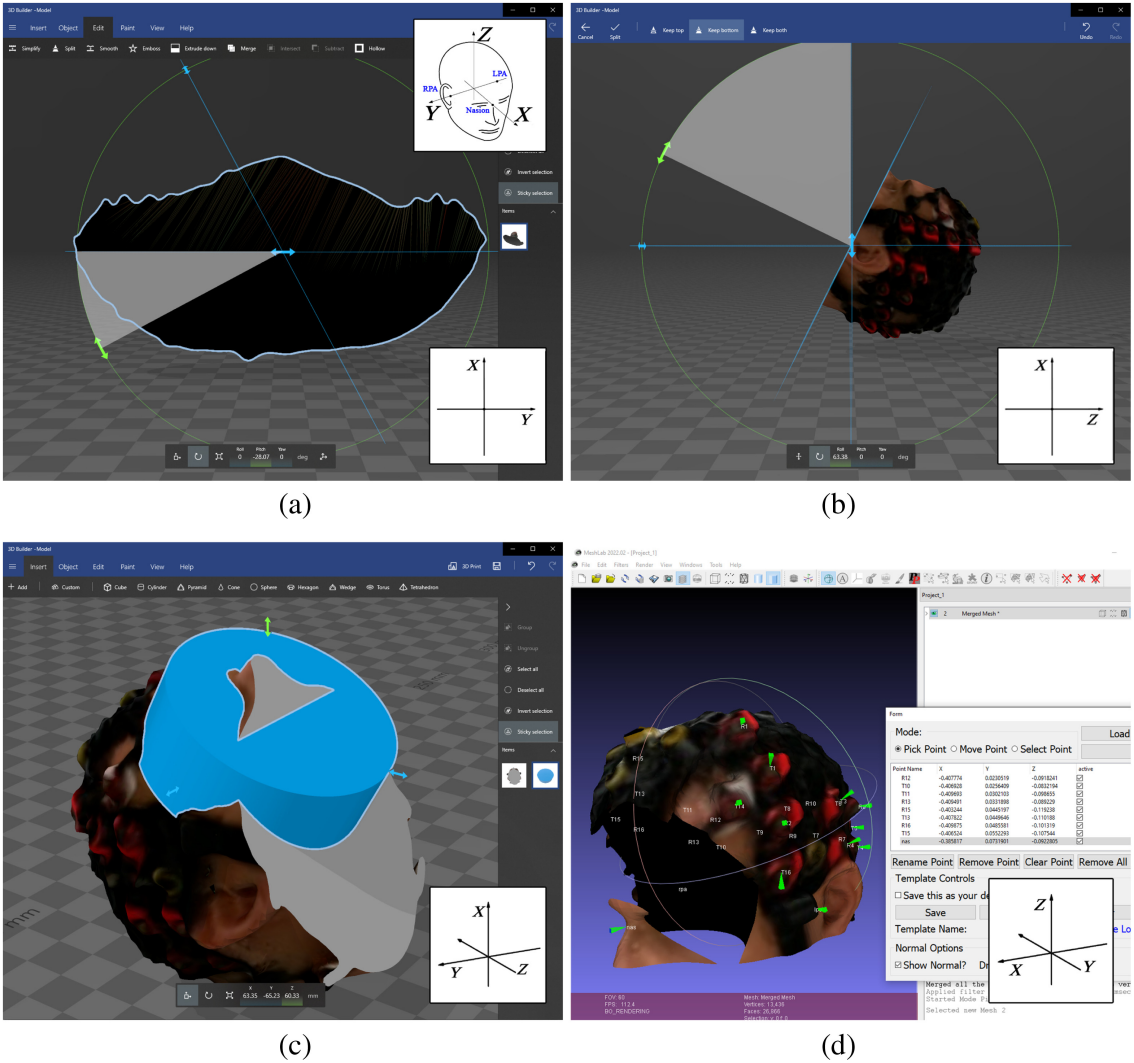
Processing of 3D Mesh. (a) In 3D Builder, we rotated the mesh along the Z-axis to place the nasion on the X-axis. The head coordinate system is shown in the top right corner, and the applied orientation is shown in the bottom right corner of each step. (b) Using the split tool, we removed the lower half of the mesh. (c) We placed a cylinder to cover the face while preserving the necessary anatomical landmarks. (d) We labeled the anonymized mesh in MeshLab. We placed markers on the head using the PickPoints tool, with the resulting coordinates and labels listed on the right.

The faces in the images captured by the 3D infrared camera were anonymized after scanning. This avoids any workaround to preserve participant confidentiality during the scanning procedure, such as covering the face or wearing a mask. For this process, we utilized 3D Builder (Microsoft Corporation, Redmond, Washington, United States, ver. 20.0.4.0). We imported the mesh files (.obj, .mtl, and .png) and kept the default unit of the files, and if the software offered it, we let it repair the mesh. In the first step, we oriented the mesh into a transverse view and rotated along the Z-axis until the nasion was on the X-axis [Fig. 2(a)]. Next, in a sagittal view, we used the split tool to remove the lower half of the mesh. We set the cutting plane to retain visibility of essential anatomical landmarks (nasion, LPA, RPA) and their proximities [Fig. 2(b)]. A cylinder was then added, positioned, and reshaped to cover the remaining face while preserving anatomical landmarks. Once the cylinder was correctly positioned, we used the subtract tool to remove the cylinder from the main mesh [Fig. 2(c)]. We saved the final mesh in .glb format to retain color information.

It is advisable to decide on the anonymization step beforehand as the anonymized mesh has a different coordinate system than the original mesh, necessitating subsequent adjustments in the labeling.

### 2.3 Labeling of Optode Positions and Data Processing

We utilized MeshLab (ver. 2022.02) for labeling of the anonymized meshes.^14^ After importing the mesh, we applied the “Flatten Visible Layers” filter from the Filter/Mesh Layer menu. Next, we used the PickPoints tool to place markers at the optode positions, as well as at the nasion, LPA, and RPA. When using the PickPoints tool, it is possible to orientate the mesh by pressing the “not editing” button. The placed markers appear in a list on the right side of the screen, with the “point name” column available for naming them accordingly. At the end, we saved the labeled markers in .pp format [Fig. 2(d)]. In instances when MeshLab froze while loading the mesh, creating a new anonymized mesh resolved the issue.

After the labeling was finished, we imported the list of optode positions into Python. We then selected the standard 10-05 montage from the MNE-Python (ver. 1.0.3) library as the template for alignment.^11^^,^^12^ Then, we performed a rigid registration of the optode positions over the standard 10-05 positions, using control-point mapping. We used two different alignment approaches:

Landmark-based alignment: We selected the nasion, LPA, and RPA as control points. This approach aligned the optode positions in relation to the participants’ anatomy. This is a robust estimate of accuracy as it represents realistic experimental or clinical conditions.

Landmark-free alignment: As an alternative method, we removed the anatomical landmarks and used all points as control points. This approach was intended to eliminate the influence of anatomical landmark positioning and provide system-specific details.^6^

After rigid registration, the positions were in Montreal Neurological Institute (MNI) coordinates, prepared for use in the MNE-Python framework for further processing.

### 2.4 MRI Registration

For each participant in the study, we conducted a three-Tesla MRI scan using a Prisma scanner (Siemens, Erlangen, Germany). The procedure included an isometric, high-resolution T1-weighted scan with the following parameters: MPRAGE with TR = 2300 ms, TE = 2.96 ms, and TI = 900 ms, and a voxel size of 1  mm×1  mm×1  mm.

We labeled the MRI scans in Slicer (ver. 5.4.0). The nitroglycerin capsules appeared as high-intensity signals on the T1-weighted MRI images. In one case, an artifact obscured the LPA point, and its position was estimated from the post-scan picture. To preserve participant confidentiality, we converted the original DICOM images to NIFTI format, which does not contain any subject-identifying metadata. The DL+DiReCT library was then used for brain extraction and segmentation.^15^^,^^16^ On the anonymized data, the segmented brain and the position of the nitroglycerin markers were shared.

### 2.5 Data Analysis

To evaluate the accuracy of the infrared 3D registration procedure, we took the points obtained by MRI as a reference. The registered positions were aligned in the MNE-Python library, and we used the Euclidean distance (in MNI coordinates) as the primary metric for error calculation.

First, we calculated the distance of the nitroglycerin markers between each participant’s MRI scans. We consider this as the errors arising during *standard cap fixation*, when the fNIRS cap is positioned according to the EEG system, and no spatial registration is performed. In this case, the effect of head circumference and head shape influences the mounting of the fNIRS cap. Second, we calculated the distance between the MRI-based positions and those from consecutive 3D scans, first within the subject and then averaged across all participants. This gave us the accuracy of the infrared 3D registration method. Third, we calculated the distance between consecutive infrared 3D scans, first within the subject and then averaged across all participants, thereby determining the reproducibility of the infrared 3D registration method. Finally, we computed the inter-optode distances on the labeled scans, using both MRI-based and 3D scan-based data. We used T-tests for the statistical analysis of the derived parameters.

## 3 Results

The inter-optode distance on the cap was fixed at 30 mm. The 25 optodes were connected to form a total of 24 channels for analysis. For five participants, it meant 125 data points for MRI-based scans and 375 data points for the infrared 3D scans, as it was repeated three times. The average head circumference of the participants was 56.6 cm with a standard deviation (SD) of 2.9 mm and a range of 54 to 61 cm. First, we analyzed the positioning of the nitroglycerin markers across participants (i.e., accuracy of standard cap fixation). On average, the Euclidean distance of these markers among different participants was 15.25 mm (SD = 8.0 mm), with the largest variance observed at optode “D1” which had a maximum error of 43 mm among participants. The accuracy of the infrared 3D registration was evaluated by comparing the infrared 3D registered positions with the reference positions obtained from the MRI scans. The average error with the landmark-based alignment was 5.69 mm (SD = 1.73). This error is significantly smaller than with standard cap fixation (p<0.001). The individual participants are visualized in Fig. S1 in the Supplementary Material. Using the landmark-free alignment, the error was 2.55 mm (SD = 1.01). The reproducibility of the infrared 3D registration process was evaluated by comparing the positions from consecutively performed infrared 3D scans. The average error with the landmark-based alignment was found to be 3.43 mm (SD = 1.62), and that with the landmark-free alignment was 1.72 mm (SD = 0.77). The average inter-optode distance for the selected channels was 29.28 mm (SD = 1.12) based on the MRI scans and 29.43 mm (SD = 1.96) based on the infrared 3D scans.

The acquisition of a single 3D mesh with the infrared 3D scanner takes 2 min, the reconstruction is almost instant, and the labeling process with a certain level of experience takes less than 5 min.^7^^,^^17^ The results with the landmark-based and landmark-free alignment are summarized in Table 2.

**Table 2:** Registered optode positions.

Name	X (SD)	Y (SD)	Z (SD)	Standard cap fixation	3D registration using landmark-based alignment		3D registration using landmark-free alignment	
				Accuracy	Accuracy	Reproducibility	Accuracy	Reproducibility
D1	−54 (1.63)	12 (15.24)	67 (4.25)	19.91 (15.36), 43	7.06 (1.88)	4.02 (1.97)	2.5 (0.84)	1.46 (0.61)
D10	−3 (3.24)	−127 (5.82)	25 (11.27)	18.29 (9.68), 36	5.53 (2.5)	3.87 (0.94)	2.4 (1.05)	1.47 (0.43)
D12	76 (1.5)	−78 (4.69)	20 (5.61)	11.18 (3.78), 18	4.83 (1.05)	2.55 (1.43)	1.75 (0.76)	1.46 (0.54)
D13	85 (3.89)	−53 (4.65)	1 (3.27)	10.13 (3.99), 19	6.4 (1.16)	2.45 (0.86)	2.37 (0.85)	1.11 (0.53)
D15	57 (2.17)	6 (12.7)	67 (5.58)	18.8 (11.81), 36	7.08 (1.63)	3.51 (1.72)	3.56 (0.92)	1.76 (0.62)
D16	78 (1.64)	3 (6.32)	15 (8.83)	16.21 (6.2), 26	6.17 (0.99)	2.54 (1.37)	2.45 (1.08)	1.97 (0.72)
D2	−73 (1.14)	7 (8.78)	14 (7.18)	15.1 (9.84), 30	6.51 (1.56)	3.82 (1.97)	2.23 (0.9)	1.59 (1.13)
D4	−83 (3.37)	−51 (5.12)	−4 (2.62)	9.92 (3.55), 16	5.31 (1.83)	3.54 (1.58)	2.29 (1.14)	1.52 (0.73)
D6	−79 (3.13)	−77 (7.2)	23 (5.74)	14.09 (6.16), 23	5.4 (1.59)	4.8 (2.55)	2.39 (0.93)	2.0 (0.73)
D7	−30 (4.08)	−126 (2.32)	−6 (10.3)	15.51 (8.91), 33	4.6 (2.34)	3.53 (1.78)	2.89 (1.01)	1.73 (1.12)
D9	26 (2.4)	−127 (4.84)	−6 (10.5)	17.19 (7.28), 33	5.28 (2.8)	2.8 (1.39)	3.34 (1.34)	1.06 (0.57)
S1	−69 (0.95)	7 (12.29)	42 (5.62)	17.65 (12.14), 36	6.21 (1.85)	3.58 (2.3)	2.13 (0.72)	1.6 (0.63)
S10	78 (3.62)	−80 (5.55)	−9 (4.84)	12.55 (3.29), 19	6.11 (1.39)	2.39 (1.28)	1.92 (0.81)	1.6 (0.9)
S11	81 (1.61)	−51 (5.93)	29 (3.19)	10.0 (4.46), 19	5.59 (1.31)	2.94 (1.72)	2.97 (1.9)	1.97 (1.77)
S13	72 (1.38)	4 (9.41)	43 (7.38)	17.04 (8.48), 29	6.22 (1.86)	3.48 (1.28)	2.13 (0.64)	1.68 (0.47)
S14	−61 (1.36)	32 (10.74)	24 (8.67)	17.37 (13.38), 37	6.45 (1.21)	3.59 (1.67)	2.79 (1.21)	2.35 (0.89)
S15	66 (1.76)	28 (7.88)	23 (10.99)	19.66 (8.86), 34	5.74 (0.99)	3.58 (1.31)	1.96 (0.75)	2.23 (0.65)
S16	−73 (1.96)	4 (5.07)	−15 (7.87)	12.41 (8.64), 25	5.79 (1.49)	2.72 (1.62)	2.45 (1.1)	1.29 (0.4)
S3	−80 (2.36)	−49 (8.38)	25 (2.56)	12.96 (6.16), 23	4.94 (1.47)	4.21 (1.69)	2.75 (0.77)	1.71 (0.57)
S4	−78 (3.43)	−80 (4.13)	−6 (5.47)	11.25 (4.51), 17	4.73 (1.6)	3.52 (1.65)	2.87 (1.19)	1.68 (0.87)
S5	−55 (3.92)	−111 (3.41)	2 (10.54)	15.65 (10.01), 34	5.57 (2.07)	3.68 (2.3)	2.66 (1.11)	2.26 (0.84)
S6	−31 (3.88)	−122 (5.95)	23 (10.92)	17.92 (10.16), 36	5.47 (2.88)	4.07 (2.33)	2.68 (1.17)	2.0 (1.4)
S7	−2 (3.27)	−131 (3.88)	−5 (10.74)	16.97 (8.03), 34	4.96 (2.03)	3.67 (1.33)	2.94 (0.77)	1.81 (0.66)
S8	26 (2.02)	−125 (4.76)	23 (11.28)	17.58 (8.73), 34	4.79 (2.04)	3.31 (1.16)	2.48 (1.19)	1.67 (0.79)
S9	52 (1.14)	−114 (4.73)	3 (9.77)	15.89 (6.73), 29	5.59 (1.62)	3.55 (1.29)	2.85 (1.23)	1.93 (0.65)
Average distance in millimeters:				15.25 (8.0)	5.69 (1.73)	3.43 (1.62)	2.55 (1.01)	1.72 (0.77)

Name: The name of the registered optode.

X, Y, Z (SD): Average and standard deviation (SD) of the registered MNI coordinates based on MRI in millimeters.

Accuracy of standard cap fixation: Average, SD, and max distance of registered MNI coordinates. Distances were calculated from the comparison of MRI scans among different participants.

Accuracy of 3D registration: Average distance and SD of registered MNI coordinates. Distances were calculated between the MRI and infrared 3D scans, first within-subject and then averaged over all participants.

Reproducibility of 3D registration: Average distance and SD of registered MNI coordinates. Distances were calculated among three successive infrared 3D scans, first within-subject and then averaged over all participants.

## 4 Discussion

In this technical note, we presented a method for performing spatial registration of probe positions for neuroimaging applications. Such improved mapping is pivotal, especially in optical measurements, where the fixed inter-optode distance poses challenges with the alignment of standard EEG landmarks. The utilized infrared 3D scanner is commercially available, has been applied in various healthcare applications, is cost-effective, and requires only an iPad to operate. It is also simple and easy to use, allowing 3D models of objects to be captured by simply walking around them, with an acquisition time of 2 min and almost instant reconstruction of a single 3D mesh.^6^^,^^17^

We aimed to evaluate the system under realistic experimental or clinical conditions. Therefore, we tested on human participants, and we used MRI scans to determine the precise positions of optode holders, which served as our reference for evaluation.

First, we evaluated the accuracy of different cap fitments by comparing the registered positions among participants based on MRI. This error is particularly important for studies that do not include spatial registration. In these cases, the measurement cap is typically placed according to the EEG system, and it is assumed that the measurement channels cover the corresponding EEG positions. However, this can never be achieved due to the fixed inter-optode distance. The average Euclidean distance between participants was 15.25 mm (SD = 8.0), with one optode showing a maximum error of 43 mm (i.e., the accuracy of standard cap fixation, Table 2). This large optode displacement, if uncorrected, could lead to misinterpretation in the analysis, as these channels might cover regions that have different signal characteristics and different functions.^4^^,^^18^^,^^19^

Then, we determined the spatial accuracy of the infrared 3D scanning method by comparing its results with the MRI-derived reference points. By performing this comparison using landmark-based alignment, we aimed to provide a robust estimate of accuracy, as optode positions are determined in relation to anatomical landmarks. This comparison yielded an average error of 5.69 mm (SD = 1.73) (i.e., the accuracy of 3D registration using landmark-based alignment, Table 2). The spatial registration provides a significant improvement, compared with the standard cap fixation. Comparing our method with other, even closely related, methods is challenging due to differences in the determination of ground truth, the number of participants, and the number of anatomical landmarks used, all of which affect the final accuracy. Nevertheless, methods using comparable or even more accurate ground truth information reported slightly higher errors using landmark-based alignment both for photogrammetric and electromagnetic techniques (Table 3). An overview of previously reported methods can be found in Table S1 in the Supplementary Material. Taberna et al.^21^ reported the Euclidean distance between the detected electrode positions and their closest points on the MRI-based head shape, rather than MRI-derived reference points, making direct comparison difficult. Jaffe-Dax et al.^7^ used reference points and reported a smaller error of 3.4 mm. The reason for this is that they used additional landmarks mounted on the head and cap, therefore allowing a non-rigid registration (i.e., deformation of the head shape). However, this approach requires additional cap preparation time for marker placement, and due to the photogrammetric approach, the reconstruction time constrains the immediate feedback of the recording.^6^ The advantage of our method is the immediate reconstruction and the use of visible landmarks on the head, eliminating the need for any additional markers. Notably, the SD of our method’s accuracy (1.73 mm) was lower than expected with the standard EEG cap (5.5 mm, as per Ref. 3). This suggests that the infrared 3D registration method can support not just fNIRS but also EEG studies by offering improved spatial accuracy.

**Table 3:** Accuracy of different registration methods.

	Xia et al.^6^	Jaffe-Dax et al.^7^	Homölle and Oostenveld^17^	Hu et al.^8^	Koessler et al.^20^	Taberna et al.^21^	Our method
Accuracy with landmark-based alignment	9.3 to 9.5 mm	3.4±0.9 mm	N.A.	6.66 mm	N.A.	1.75 mm	5.69±1.73 mm
Accuracy with landmark-free alignment	1.8 to 2.6 mm	N.A.	9.4 mm	N.A.	2.11 mm	N.A.	2.55±1.01 mm

To provide further insights into the accuracy of the system and to ease the comparison with other methods, we conducted additional analyses:

i. We isolated the error from the anatomical landmark positioning, by performing a landmark-free alignment (Table 2). It resulted in an average error of 2.55 mm (SD = 1.01), which is comparable to a recent report conducting a similar analysis.^6^
ii. We calculated the error between the consecutively performed infrared 3D scans (i.e., reproducibility of 3D registration, Table 2), which is consistent with prior research.^7^
iii. We recalculated the fixed 30 mm inter-optode distances. This error is independent of the ground truth information, and the only source of error is the uncertainty in the acquisition method and the labeling, although it reflects the accuracy only for optodes that are spatially close to each other. The errors based on the MRI scans and the infrared 3D scans both reached the lower limit of the resolution of their respective acquisition methods.^22^

In conclusion, our study presents a comprehensive solution for integrating spatial registration into neuroimaging research protocols. We demonstrate the use of a commercially available device for measurements and 3D reconstructions, along with guidelines for data anonymization and labeling. The acquired positions can be loaded and processed in the toolbox of choice. We provided a solution for MNE-Python integration. The scripts to reproduce these results are freely available at Ref. 23. To apply to individual data, researchers need to save their optode positions into a .pp file for each participant (described in Sec. 2.3) and specify the channels used in their unique montage in the script.

### 4.1 Limitations

In our study, we only measured five participants. The range of head sizes in our sample ranged from small (min 54 cm) to very large (max 61 cm). For individuals with very small or extremely large head sizes, there would likely be even more variability in the optode positions. Although the number of participants is limited, the S to XL size range covers the majority of the population. Searching for participants outside of this range would be an unjustified effort, resulting in excessive use of the MRI scanner with minimal expected benefit. Furthermore, we limited ourselves to an inter-optode distance of 30 mm, which is an often-used distance in fNIRS research.^4^^,^^10^^,^^24^^,^^25^

### 4.2 Conclusion

For optimal neurophysiological assessments, it is imperative to ensure precise probe localization. Utilizing a 3D infrared scanning technique for spatial registration demonstrated economic efficiency, simplicity, repeatability, and high accuracy. In addition, we provide practical guidance on anonymization, labeling, and post-processing of acquired scans, making them suitable for academic use. The acquired infrared 3D and MRI scans, together with the source code for reproducing the results and using the positions in the MNE-Python library, are available in the linked data repository.

## Supplementary Material



## Data Availability

The measurement data and the processing scripts are available on Dryad: https://datadryad.org/stash/share/gh1nSH-zPMSWB73OC2YV4uvrbLo54xJjgmfVb3M2xEA (Ref. 23).
