# Supplementary material for: Three-dimensional infrared scanning: an enhanced approach for spatial registration of probes for neuroimaging
Source: Neurophotonics. 2024 May 26;11(2):024309. doi: 10.1117/1.NPh.11.2.024309 (PMC11134420; doi:10.1117/1.NPh.11.2.024309)
Supplement: Supplementary file 1 [file NPh_011_024309_SD001.pdf]

Supplementary information

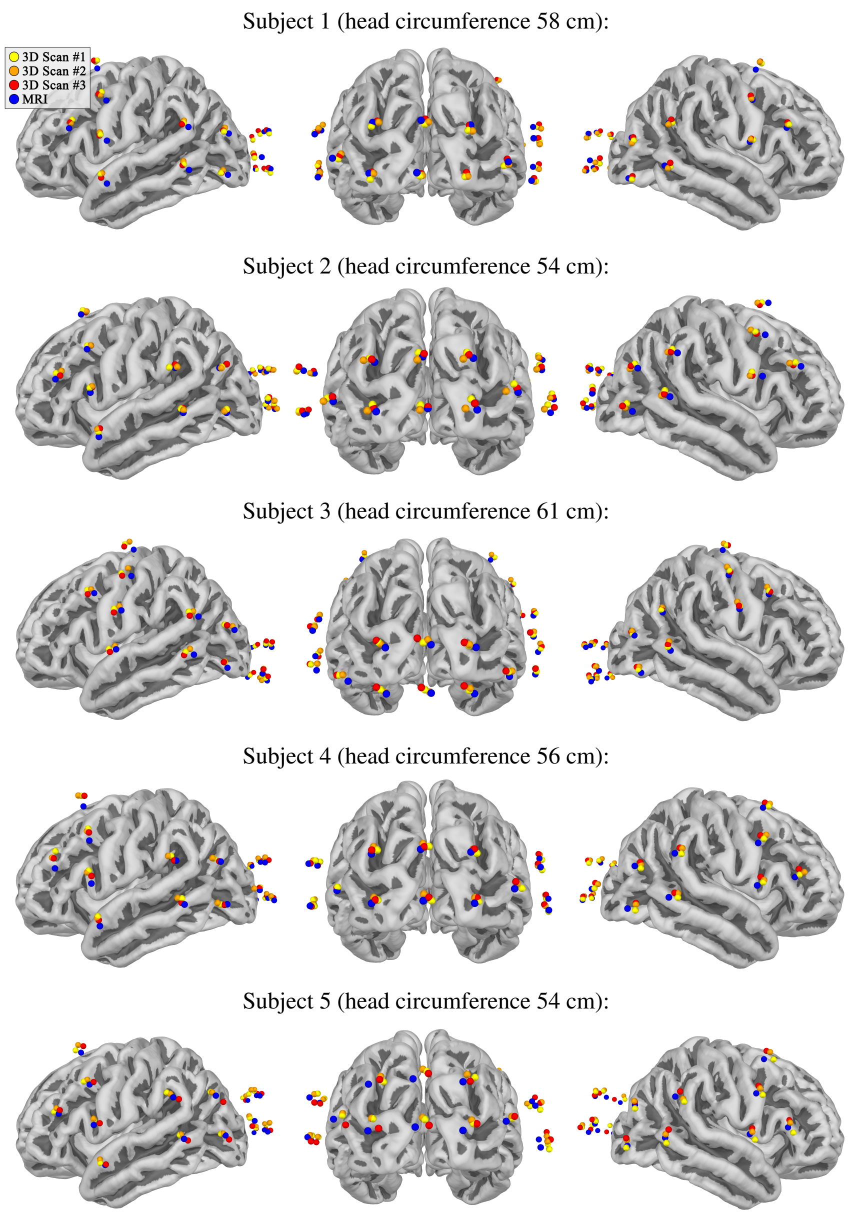

Figure S1: Visualization of spatial registrations after landmark-based alignment, in the MNE-Python library. The left columns is the lateral, the middle is the caudal and the right is the medial view. We marked the registered positions with different colors, according to the registration method.

Table S1: Detailed literature comparison

	Xia et al., 2023	Jaffe-Dax et al., 2020	Homölle et al., 2019	Hu et al., 2020	Koessler et al., 2011	Taberna et al., 2019	Our method
**Scanning method**	Unique Photogrammetry	Photogrammetry	IR scanning	Photogrammetry	Laser	IR scanning	IR scanning
**Hardware**	5 mobile phone cameras, attached to a handcrafted hollow aluminium loop and controlled by a custom PCB	GoPro	iPad + Structure Sensor	iPad	3D Laser scanner	iPad + Structure Sensor	iPad + Structure Sensor Pro
**Hardware commercially available**	No	Yes	Yes	Yes	Yes	Yes	Yes
**Cost effective**	Yes	Yes	Yes	Yes	No	Yes	Yes
**Ground truth**	3D-printed CAD model	Electromagnetic digitizer	Electromagnetic digitizer	MRI, slice = 1.3 mm	MRI, slice = 1.2 mm	MRI, slice = 1.2 mm	MRI, slice = 1 mm
**Cap**	Full head fNIRS cap	Full head fNIRS cap	Full head EEG cap	Unilateral fNIRS cap	Full head EEG cap	Full head EEG cap	Full head fNIRS cap
**Free anatomical landmarks**	Nasion, LPA, RPA	Nasion, LPA, RPA	not used	Nasion, LPA, RPA	Nasion, LPA, RPA + Face Modeling	not used	Nasion, LPA, RPA
**Anatomical landmarks covered by fNIRS cap**	Inion, Cz	Inion, Cz, Pz, front, left and right side of the cap	not used	Inion, Cz	not used	not used	not used
**Scanned points**	99	139	59	18	64	128	25
**Subjects**	1 infant Phantom	10 adults	50 adults	22 adults	4 adults	8 adults	5 adults
**Quick enough scanning time for infants**	Yes	Yes	No	Yes	No	No	No
**Immediate reconstruction**	No	No	Yes	No	N.A.	Yes	Yes
**Anonymization**	No	No	No	No	No	No	Yes
**Code fully available**	No	No	Yes	No	No	No	Yes
**Alignment Method**	Point based rigid registration	Point based registration, with additional deformation of head model during alignment	Point based rigid registration	Point based rigid registration	Face mapping to MRI	Head shape mapping to MRI, then point based rigid registration	Point based rigid registration
**Evaluation**	Median Euclidean distance error between the registered positions and true positions<br><br>Comparison with:<br>• Photogrammetry<br>• Electromagnetic digitizer (Polhemus)<br><br>Measurements in static and moving head scenarios	Mean Euclidean distance error between the registered positions and true positions	Median Euclidean distance error between the registered positions and true positions	Mean Euclidean distance error between the registered positions and true positions	Mean Euclidean distance error between the registered positions and true positions	Mean Euclidean distance error between the registered positions and their closest points on the MR-based head shape<br><br>Comparison with:<br>• Electromagnetic digitizer (ANT Neuro)	Mean Euclidean distance error between the registered positions and true positions
**Accuracy with landmark-based alignment**	proposed method:<br>• 9.3 mm (static)<br>• 9.5 mm (moving)<br>Photogrammetry:<br>• 10.0 mm<br>Electromagnetic digitizer:<br>• 9.0 mm	3.4 ± 0.9mm	N.A.	6.66 mm	N.A.	N.A.	5.69 ± 1.73 mm
**Accuracy with landmark-free alignment**	Proposed method:<br>• 1.8 mm (static)<br>• 2.6 mm (moving)<br>Photogrammetry:<br>• 2.2 mm<br>Electromagnetic digitizer:<br>• 3.7 mm	N.A.	9.4 mm	N.A.	2.11 mm	Median of all caps (Suppl. Table 3)<br>Proposed method:<br>• 1.75 mm<br>Electromagnetic digitizer:<br>• 2.54 mm	2.55 ± 1.01 mm
**Reproducibility**	N.A.	2.0 ± 0.5 mm	N.A.	N.A.	N.A.	N.A.	3.43 ± 1.62 mm

* CAD: Computer Aided Design, CZ: Midline Central EEG point, EEG: Electroencephalogram, fNIRS: Functional Near-Infrared Spectroscopy, IR: Infrared, LPA: Left Pre-Auricular, MRI: Magnetic Resonance Imaging, PCB: Printed Circuit Board, RPA: Right Pre-Auricular
